# Hospital and Institutional News

**Published:** 1915-04-24

**Authors:** 


### HOSPITAL AND INSTITUTIONAL NEWS.

### THE NEW RED CROSS COMMISSION.

The Joint War Committee of the British Red Cross Society and the Order of St. John of Jerusalem have determined to send a Commission to Malta and the East. Under the approval of the Admiralty and the War Office, Lieutenant-Colonel Sir Courtauld Thomson has been appointed Chief Commissioner, and is expected shortly to take up his new duties in the Mediterranean. It will be recalled that Sir Courtauld Thomson has already been actively engaged in Red Cross work. The first Commission, which went to Belgium, had Sir Alfred Keogh as its Chief; and when he went to the War Office in the autumn Sir Arthur Sloggett became Chief Commissioner. On Sir Arthur's proceeding to Headquarters, Sir Courtauld Thomson went out as Commissioner to Boulogne, and there he engaged in such a strenuous period of work and investigation that he was compelled to withdraw, temporarily as his new appointment shows, from these duties. Sir Arthur Lawley, who has been acting in Sir Courtauld Thomson's absence, succeeds him as Commissioner in France.

### DEATH OF A GREAT CHAIRMAN.

The death of Mr. John Marshall, chairman of the board of management of Sheffield Royal Infirmary, removes a great institutional manager, who devoted his best energies to the important post which he held for twenty-two years. But it was for the period of thirty-seven years that Mr. Marshall actively associated himself with the affairs of his institution, and his length of service has only once been surpassed, namely, when one of his predecessors, Mr. James Montgomery—who was also, by the way, a poet—was chairman for twenty-five years. Indeed, the value of his public work was such that we felt an account of it merited publication in The Hospital. Accordingly, in our issue for April 27, 1912, an appreciation was published in the series on "Eminent Chairmen" that was then appearing in our columns. To that our readers may turn for details of his work. We may, however, recall here that he joined the hospital board in 1875 and succeeded the late Mr. Brooksbank as chairman in 1890. Mr. Marshall was noteworthy in that the scientific as much as the financial side of the Royal Infirmary appealed to him, and its equipment to-day is largely a monument to his energy and enthusiasm. He was able as chairman to hold, with nice adjustment and skill, the balance between administrative and medical interests, and the happy relations of the different staffs was at once his success and his reward. Naturally, in such a period and under such control, the work and developments of the institution have grown enormously, and Mr. John Marshall will be remembered as one of the pillars of that voluntary system, whose chief glory it is to avail itself of such personal service as he willingly placed at the disposal of the Sheffield Royal Infirmary. Presentations were made to him by members of the board in 1906 and in 1913. His portrait hangs in the board room.

### WOMEN AS HOSPITAL PORTERS.

What seems likely to prove a successful scheme has just been started at the Royal Hampshire County Hospital, Winchester, and it is possible the suggestion may prove useful to other provincial hospitals where there is difficulty in obtaining a sufficient number of porters to fill the places of those who have left for service in the Army or Navy. By rearranging the various duties of the hall porters and dispensary porter it was found that a great deal of the lighter work could easily be undertaken by women. As soon as the need for such help was made known several young women offered their services and were accepted. Their hours of work are short—not more than five hours a day, and they have quite finished by 6.30 p.m. Two work in the dispensary, taking alternate fortnights on duty. Others attend to the telephone and perform the usual duties of a hall porter. The wages of the men whose places they fill are divided between them. They wear a simple dark uniform. The plan is understood to be working smoothly and well, and the women's work, we learn, is very well done.

### THE DIFFICULTIES OF SECTIONAL REPRESENTATION.

From the columns of the local Press we gather that one of two Leicester Trades Council representatives on the Board of Governors of the infirmary was not reappointed as a member of the house committee of that institution, and that at a recent meeting of the Trades Council the member in question referred at length to his exclusion and urged the Council to "fight the matter, or withdraw from the infirmary," further expressing the opinion that what they wanted to do at the present time was to "stop money going to the infirmary," and that the section of trade which he represented "would back up a movement to withhold contributions to the infirmary until he was reinstated." The above expressions are self-condemnatory, and one of the members of the Council remarked at the meeting in question that the Trades Council were unhappy in their choice of this particular representative on account of his want of tact and judgment. We should, however, like to point out to working men representatives the attitude of hospital managers, which is this. Hospital boards and committees are convened for the transaction of institutional business; if sectional representation is a hindrance to business and organisation, it becomes a definite discouragement to busy and experienced professional and commercial men, who not only give valuable time, but take great pride in assisting in the many administrative details of a large hospital such as the Leicester Royal Infirmary. The Leicester Trades Council will be best serving their own interests, and the interests of the 20,000 or so working men affiliated with them, by nominating a representative whose views harmonise with the majority of the members of the Council. It is clear that sectional representation is a failure when controversy and distorted statements are habitually introduced, to the checking of routine and method. Moreover, the administrative details for the care of 300 sick and suffering fellow-creatures are so complex and difficult that irregular criticism is out of place at hospital committee meetings, which can only be conducted by men fully instructed in the tenor and details of hospital affairs. Such instruction will not prevent men from speaking out when they believe criticism to be advisable, but will lend weight to their remarks from the restraint which is the fruit of knowledge.

### THE LOSS OF SUBSCRIBERS THROUGH ENLISTMENT.

The Committee of the Royal Infirmary and Children's Hospital, Sunderland, lately issued the following telling appeal in the county newspapers: "The committee of [the two] institutions earnestly appeal for subscriptions and donations to make up loss of revenue amounting to £120 a month, due to the fact that thousands of weekly contributors have joined the Navy and Army." The hospital's secretary is Mr. Thomas Robinson, and last week he received on behalf of his institution a cheque for one thousand guineas from Mr. R. A. Bartram, J.P., a veteran supporter of the institution. In enclosing the cheque, Mr. Bartram expressed his appreciation of the hospital's work, and said that he felt sure that Mr. Robinson's own services had largely contributed towards it. We have described Mr. Bartram as a veteran supporter, and we need only recall here the fact that he lately contributed £1,000 towards the fund being raised for the reconstruction of the administrative block at the Royal Infirmary. We hope that his example will aid the appeal, and produce other gifts as generous as the one here recorded.

### THE LOCAL PRESS AND THE MONTHLY MEETING.

Surely the sorriest columns in the provincial newspapers are those which record with dreary detail and at elaborate length the purely routine business which is got through as quickly and formally as possible by the members who attend meetings of local public bodies. No doubt the local paper has to be filled. No doubt local news is that with which it should concern itself. No doubt local bigwigs are of more interest to a local editor and his readers than Kings and Queens or Prime Ministers elsewhere. But there is surely no reason why this healthy natural interest in local affairs should have no saving grace, and indeed be degraded and made ridiculous. An account lies before us, as we write, of a column in length, concerning the procedure of a certain hospital board which illustrates the pompous absurdity with which the proceedings of these necessary bodies may be conducted. A list of names and titles heads the account, and the notabilities present may be gauged by the fact that half the letters of the alphabet follow the surnames of each member. On examination these designations resolve themselves into such descriptive summaries as the following: J.P., R.D.C., U.D.C., T.C.—in short, with the exception of the first, they refer to the different committees for local affairs to which each member happens to belong. It is out of observations of this kind that the comedies of Molière and the novels of Dickens were written. In the present case the proceedings were fully worthy of this inflation. Apart from a letter making a request in relation to insurance, the annual report of the local institution was considered; that is to say, no single point in it was discussed or even mentioned, but members commented at length upon its interest and value, and complimented the medical officer on the skill with which it had been drafted. For these compliments the medical officer then expressed his thanks. A monthly report was submitted, and the annual estimate discussed. The question of a loan was raised; an appointment, for which there was only one applicant, was made; it was decided, after discussion, not to change the hour of meeting; and, finally, the date of the annual election of members of the board was shown to be such that some of the members must be disqualified. It is surely fitting that a column headed with such pomposity should fizzle out in such a manner at its end.

### A SUPPORTER OF HOSPITALS IN SOUTH WALES.

The freedom of the City of Cardiff has been conferred upon Sir William James Thomas in recognition of his great munificence. Every Christmas he doubles the amount of relief granted in his district. He has endowed a bed at the Porth Cottage Hospital, maintains one of its wards, and is an annual subscriber. A little over a year ago he subscribed £1,000 towards the funds of the Royal Gwent Hospital at Newport, and his gifts to King Edward VII.'s Hospital, Cardiff, amount to about £18,000. From time to time he has subscribed to the funds of the University College of South Wales and Monmouthshire, of which he is one of the governors, and has crowned his benefactions by giving £90,000 to the fund for the medical school, thus making certain both its creation and an annual maintenance grant from the Treasury. Ambulances, churches, and chapels also owe much to his financial assistance.

### NEW CHAIRMAN OF WOOD GREEN HOSPITAL.

The Passmore Edwards Hospital at Wood Green, one of the several institutions in Greater London associated with this name, has appointed a new chairman of the Council in succession to Mr. Harry Smith. Mr. G. T. Brown, who has been elected to the post, has been very closely associated with the affairs of the hospital for many years—in fact, since it was first established in 1895. He has, therefore, in the many capacities in which he has served, gained an inside working knowledge of its affairs, and been concerned in the two enlargements that have occurred in the last five years. The vice-chairman of the Council is Dr. Ferguson, and the chairman of the house committee is Dr. Dickson.

### WORKHOUSE MEDICAL OFFICER AND FEES FOR OPERATION.

District medical officers are entitled by Article 177 of the General Consolidated Order, 1847, to fees for the performance of certain specified operations. In the institutional letter issued with the Order the Commissioners stated that the workhouse medical officer was excluded because they thought important surgical operations should not be performed in workhouses, and that if they had to be performed the subsequent necessary attendance would not be such a severe burden upon him as in the case of the district medical officer. The first of these reasons no longer exists, since the Local Government Board encourages rather than discourages the performance of operations in Poor-Law institutions; for the second there are some grounds; but it must be remembered that after an operation special visits are often required. The part-time medical officers of Poor-Law institutions have, therefore, a grievance in the matter, and this is recognised by the Malden Guardians, who have decided to ask the Local Government Board for sanction to pay their workhouse medical officer the same operation fees as are payable to district medical officers. The result of the application will be awaited with interest by other medical officers in a similar position.

### AN INFIRMARY BOOK-KEEPING CASE.

A curious discrepancy in the keeping of the provisions account in connection with the Archway Road Infirmary was considered at a meeting of the Holborn Board of Guardians last week. The visiting committee of the institution reported having ascertained that for the half-year ending on September 30 last, 3,720 eggs, representing £13 8s. 8d., were unaccounted for. After interviewing the medical superintendent, the steward, the steward's clerk, and a sister, the committee discovered that the nurses had requisitioned and been supplied with more eggs than had been ordered by the medical superintendent. It was stated by the sister that the extra unauthorised eggs had been used to make custards for the patients. The steward, while not admitting any responsibility for the alteration of the figures in the provision books, offered to pay for the eggs, and the steward's clerk had written resigning his appointment, as he felt that he had sacrificed the Guardians' confidence by altering the figures in the books. He stated that he wished to join the Colours. The committee's report went on to say that the sister in question, who admitted applying for more eggs than were ordered by the medical superintendent, had been severely censured, and informed that any departure from the medical superintendent's orders might lead to instant dismissal. The medical superintendent had been instructed to convey this resolution to the entire nursing staff. The report was adopted, and the steward's clerk was stated by the chairman of the committee to have committed an error of judgment, and to leave without a stain on his character. The committee would favourably consider an application for a testimonial. At this meeting, the three years' tenure of office of the chairman, Mr. Edward Garrity, came to an end, and he received the thanks of the board in terms which recorded the fact that he had never been absent from a single board meeting. He said in reply that during thirty-seven years of public life it had been his ambition to be chairman, and his having become so, had shown that the office was open to any humble member.

### MEDICAL OFFICERS AND CLERICAL WORK.

In a recent number of The Hospital we recorded the refusal of the Local Government Board to sanction a proposal of the Lincoln Board of Guardians to make the superintendent-nurse responsible for the filling in of the patients' record papers—a decision with which we were in entire agreement. It appears, however, that the medical officer of the Lincoln Workhouse is employed at one of the military hospitals, and cannot give the time required to carry out this clerical work. The Local Government Board has now written stating that, under the exceptional circumstances, it will withdraw its objection. The shortage of medical men is leading to many unavoidable changes in the accustomed routine of medical work. It is doubtful, however if the best solution has been found in this case. The notes of the superintendent-nurse will hardly be of much value. Some time ago, speaking of the effect of the war on medical work, we said that elaborate clinical records might have to be abandoned. In this case it would have been better to have released the medical officer from the obligations placed on him by the Poor-Law Institution Orders and left him to keep such brief memoranda of his cases as his time permitted.

### ST. GEORGE'S HOSPITAL WINS ITS CASE.

Messrs. Samuel Wallrock and Co., the firm of house agents who, as recently reported in our columns, brought an action against the president, vice-presidents, treasurers, and governors of St. George's Hospital to recover £15,000, which they claimed as due to them for commission for services rendered in connection with the proposed sale of the hospital site to Mr. Mallaby-Deeley, M.P., have lost their case. The defendants denied liability, and the plaintiffs in the alternative claimed upon a *quantum meruit*. The final hearing of the case took place before Mr. Justice Darling last week, when the plaintiffs alleged that the commission was due to them for having introduced Mr. Mallaby-Deeley to the authorities of the hospital with a view to his becoming the purchaser of the property for the sum of £525,000. Counsel for the plaintiffs argued that two questions would come before the jury: first, whether the treasurer of the hospital had authority to enter into the agreement with the firm for the payment of commission; and, secondly, if Messrs. Samuel Wallrock and Co. were not entitled to the full amount of their claim, what sum was due to them for the immense amount of work that they had done in the matter. Mr. Mallaby-Deeley in his evidence declared that the negotiations eventually fell through because he failed to come to an agreement with the Duke of Westminster concerning his rights over the estate. Counsel for the hospital submitted that on the correspondence which had passed commission was only payable if the purchase were completed and the money received by the hospital. As stated above, judgment was entered for the defendants. We congratulate the hospital on this issue of the matter, especially as the uncertainty under which the hospital's authorities were compelled to labour until the matter was determined was grave, and prejudicial to its welfare.

### SMOKING IN HOSPITALS.

So many hospitals are now counting among their patients wounded soldiers, that there is hardly an institution in the country in which, at all events in some wards, smoking is not permitted. This, of course, was rarely, if ever, the case before the war, when, if the question came up at all, as it did occasionally, it came up either as a breach of discipline or a request for permission to smoke was referred to the medical board, which would in turn forbid it, partly on administrative and partly on medical grounds. Now, however, possibly as a result of the first emotional desire to give to our wounded every single thing which we could to please them and show our appreciation of their service and gallantry, the question of reconsidering the prohibition against smoking is being raised at one or two institutions. Naturally, those in industrial districts, where the patients come from works at which, in the off-hours, smoking may be permitted, are the institutions which have received the request. We trust that wherever it is made the request will be refused. For the plain facts of smoking are obvious to anyone. While the opinion of most medical men on the matter seems to depend on whether they smoke themselves or not, it cannot be denied that it is an unnecessary habit. It is also, from the nature of the case, a dirty habit: it makes more work in the wards, it encourages spitting, and is incompatible with that rigid and detailed cleanliness which is one of the most important and valuable lessons that any hospital can teach. This being so, a practice which most of us have gladly winked at in the case of wounded men whom we delight to humour, must by no means be extended to hospital patients generally. Permission to smoke must be the special privilege of wounded, a unique concession to popular feeling, which creates no precedent, and will lapse with the war.

### DENTAL TREATMENT FOR TUBERCULOUS CASES.

The Halifax Health Insurance Committee has accepted the sanatorium sub-committee's scheme for the dental treatment of tuberculous cases, after embodying a recommendation from the Commissioners that patients should not be referred automatically to a dentist, but that only those cases which have been certified by the tuberculosis officer to require such treatment should have their teeth attended to. The amended scheme provides that any insured person whom the tuberculosis officer certifies to be in need of dental treatment, and who would not otherwise benefit from institutional treatment, shall be examined by a qualified dentist, appointed by the Insurance Committee, and no such treatment can be entered upon until the patient's teeth have been put into a proper condition. The emendation, to the acceptance of which the Commissioners' approval was attached, is perhaps open to the criticism that practically every person who is in need of institutional treatment requires to have his or her teeth attended to, and to have this secured automatically has the advantage of providing an examination in each case which cannot be guaranteed when the tuberculosis officer is not bound to include the condition of the mouth in his routine examination.

### WAR OFFICE AND THE METROPOLITAN ASYLUMS BOARD.

The War Office have again called on the Metropolitan Asylums Board to assist them in their efforts to provide accommodation for wounded and sick soldiers. The request came through the Local Government Board, with the hope from the latter authority that the managers would be able to accede to the wishes of the Army Council that the Brook Hospital might be used for the accommodation of the Forces. It was pointed out that the present urgent need for hospital accommodation would render the acquisition of this hospital by the Army Council a very desirable matter. The Hospitals Committee, who were confident the managers desired to render the military authorities all possible assistance, in suggesting that the institution should be placed at the disposal of the Army Council, were hopeful that they would not require it for more than a few months, and that it would again become available for use by the managers when the autumnal seasonal rise in fever occurs. The managers had 357 patients in the Brook Hospital, and these will be transferred to the other institution belonging to the managers. With that readiness that has characterised them through the past few months, when they took over the control of the Belgian refugees, they decided to accede to the request of the Army Council.

### WOUNDED AT RACE MEETINGS.

Some satisfaction after all, beyond the negative one of not being turned out, has been gained this week by those of the wounded who are convalescing in the Epsom and Ewell War Hospital, which, it will be recalled, is formed of an annex to the Grand Stand, and over which there was a recent controversy. The number of patients is larger than ever, including, as it does, soldiers from the local training camps, and in the Grand Stand premises on the Downs there are eighty-three patients. Through the action of Mr. H. M. Darling a box in the Grand Stand has been placed at the disposal of those of the wounded who were sufficiently well to leave their beds and were anxious to watch the races on Tuesday and Wednesday. In Mr. Langland's stands, two boxes have also been placed at the disposal of the patients. From the public point of view this is a happy ending to the affair, and those in favour of the continuance of racing will no doubt point to the pleasure which it gives to the wounded at Epsom Hospital.

### THE NEED FOR A MINISTER OF PUBLIC HEALTH.

We have lately, in these columns, suggested that the corporate bodies of the medical profession might have shown greater promptitude in giving a lead to medical men as to the best way in which their services could be arranged to meet the pressing needs of the Army for doctors, and of institutions at home where staffs have been depleted by the war. Now that something is being done in this direction it is interesting to hear that the General Medical Council is showing signs of being alive to the health problems which have culminated during the past six months, and are making representations to the Privy Council with a view to the necessity for the immediate establishment of a Ministry of Public Health. The principle of this proposal, so often made by a small band of enthusiasts, and so conveniently shelved from time to time, has always met with our warm support, and we believe that the present is a good opportunity for pressing its claims to adoption on the Government. Already the Army Medical Service, the shortage of staff in the institutions at home, the appeal for nurses, and the lack, at present, of any one central co-ordinating authority, provide the best and most apposite proof of the difficulties in which we are landed by having no Minister of Public Health to direct the isolated efforts of good will now hampered by lack of direction. The establishment of such a post is not controversial; its expediency can hardly be questioned by those who know the facts and the difficulties caused by our present lack of co-ordination. The time, therefore, is ripe for pressing forward the proposal, even if so important a change is doubtful. But if the war, which has forced these problems on us, results in the creation of this office, one valuable lesson will have been learnt, and one remarkable advance have been made for which the nation might well be thankful.

### DEATH OF A MENTAL SUPERINTENDENT.

We regret to record the death, at the advanced age of seventy-four, in Edinburgh, of Sir Thomas Clouston, M.D., late physician-superintendent of the Royal Edinburgh Mental Hospital, Morningside. Educated at Aberdeen University, he graduated M.D. in 1861, and in 1863 he was appointed superintendent of Carlisle Mental Hospital, a post which he held until he became superintendent at Morningside in 1873. The principal post of the kind in Scotland gained increased prestige during his lengthy tenure of office, owing to the way in which he negotiated the important, indeed radical, changes which were effected in mental hospital procedure and treatment during this period. He combined the rarely associated gifts of a first-rate administrator with a student's interest in science, and carried out administrative reforms and scientific investigations into mental neuroses—" The Neuroses of Development " was the title of one of his publications. To instance the thoroughness of his work in the administrative field one may recall another work of his, " An Asylum or Hospital House with Plans,"' which reveals his appreciation of institutional problems. At one time he edited the *Journal of Mental Science*, and among a host of distinctions we may recall his Presidency of the Royal College of Physicians, Edinburgh, in 1902-03. He was knighted in 1911. It is to his honour that he was one of those who turned attention to the relation between environment and mental disease; and the result was a cheerfulness and diversity in every institution, indeed in every room, which was open to his influence.

### ABANDONING THE CONSUMPTION CRUSADE.

We have already drawn attention to the number of vacant medical posts, particularly in connection with tuberculosis, which, doubtless under pressure from high authority, the local authorities are omitting at present to fill. Thus, the anti-tuberculosis crusade, on the progress of which under war conditions we lately published a special article, is now receiving a general set-back. A typical instance of this is provided by the action of the Derbyshire County Council, which has not only offered to the War Office the services of its medical staff, but has also placed at its disposal for the benefit of wounded soldiers the new sanatorium at Chesterfield. A special report was considered at the Council's meeting last week, in which it was stated that all the members of the county medical staff were willing to offer themselves for home service. The effect of the acceptance of the sanatorium would be to leave most of the anti-tuberculosis work in the county in abeyance, and therefore it will be necessary to readjust financial relations between the Council and the Insurance Committee. On the other hand, it is hoped to proceed with the dispensary work, though school medical inspection is threatened. As a matter of fact, owing to the small numbers of doctors available, it might not be possible in any case to carry on the ordinary medical services, and thus to offer the sanatorium, as was pointed out by Mr. E. C. Barnes, the chairman of the Tuberculosis Committee, was to make a virtue of necessity. Still it cannot be denied that the lapse of all these new services is a grave matter, and, we are convinced, in part an unnecessary matter, though due at present to the want of co-ordination in medical work, which is the real obstacle to its efficiency in England.

### WHAT CHILDREN CAN DO.

Acts of self-denial amongst the poor are of frequent occurrence, but since the outbreak of the war they have more than redoubled their efforts to aid those who have been wounded or are sick through their life at the Front. A delightful example of what the children are doing and can do comes from a small mission at Kennington known as "The King's Own " Mission, from its association with the Royal House through the Duchy of Cornwall estate, on which it is situated. The children at

Easter collected eggs for the hospitals, and their united efforts resulted in a total of 3,524, all newly laid, being acquired. These were distributed amongst King's College Hospital, Denmark Hill; St. Gabriel's College, Camberwell; Red Cross Hospital, Clapham; Queen Alexandra Hospital, Millbank; All Saints' Hospital, Vauxhall Bridge Road; and St. Thomas's Hospital. A pleasing feature of the distribution was that the children were permitted to take part in the distribution amongst the patients, and no doubt this fact will stimulate them to renewed efforts if they again undertake a labour of love of this kind. The personal element in these undertakings helps greatly to stimulate those who are willing to give their services at the present time; and, as the help of children is but little employed on behalf of the hospitals, we quote the above as suggestive to hospital managers in search of subsidiary sources of income.

### SUCCESSFUL CLAIM FOR WRONG DISPENSING.

Judge Parry, at the Lambeth County Court, heard, with a jury, the first claim for damages for the wrong dispensing of medicine since the introduction of the National Insurance Act. The plaintiff was a labourer named John Henry Holt, who had been suffering from facial paralysis. Dr. J. J. Rhodes, of Battersea Park Road, S.E., his panel doctor, prescribed belladonna, which was painted on the man's face, and from which he was getting gradual improvement. On May 10 he was sent with another prescription to the chemists, Messrs. Gadd & Son, of Wandsworth Road, Battersea, and was supplied with the same paint as he had used before. After applying it three days he found his face blistered, and the doctor, on examination of the bottle, came to the conclusion that his patient had been given kerosene in mistake for belladonna. The continued application of the kerosene seriously affected the man's constitution, and retarded his recovery, and it was not until the end of July that he was quite well again. He joined his regiment at the outbreak of the war as a Reservist, but was invalided out after a short time suffering from paralysis of the eye on the same side of the face on which the kerosene had been applied. The dispenser, Mr. Edgar Smith, was unable to account for the substitution of kerosene for belladonna, and although a protracted hearing of the case took place, no one was able to throw the slightest light on the mystery. Holt had made a complaint to the London Insurance Committee, and their Pharmaceutical Sub-Committee held an inquiry, when it was stated they were satisfied with the explanation offered by the chemist, though it was remarkable that the doctor who attended the man was not asked by the Committee to give his view of the matter. Though the Sub-Committee were satisfied, the jury who heard the case at the County Court were not, for they awarded Holt £40 damages and costs.

### THIS WEEK'S DRUG MARKET.

Business continues good in most departments, although the extreme prices asked for the group of synthetic drugs naturally has a restrictive effect on the demand. Carbolic acid is dearer; the Government has taken control of the production of this article, and in view of the increasingly large quantities required for making ammunition the supplies available for medicinal and disinfectant purposes are limited. There is little change in the price of cocaine, but there is an impression that a further advance is not probable at the moment and that a decline is possible, as supplies are likely to be more plentiful. The value of cod-liver oil is not maintained quite so firmly, but at the moment there is no indication of an impending fall in price of any note. Morphine and codeine are in strong request at unchanged prices. Chloroform is slightly dearer. Japanese refined camphor has an upward tendency in price. Cloves are dearer. At the recent public auction of drugs held in Mincing Lane there was a good demand for rhubarb at steady rates. Cardamoms had a lower tendency. Jamaica honey sold at higher prices. High prices were paid for senna. Sarsaparilla fetched full prices. A quantity of oil of aniseed—a naval prize—offered on account of the Marshal of the Admiralty sold at lower prices.

### COUNTY ASYLUMS AS MILITARY HOSPITALS.

We have already given an account of the conversion of the Mental Hospital at Cardiff into one for the reception of the wounded. We understand the War Office have made arrangements, which are in a forward state of completion, with the managers of some eleven of the larger county asylums in England and Wales to enable them to be taken over by the War Office as hospitals for the wounded. It is stated that the hospital accommodation to be thus provided will supply 15,000 additional beds for the wounded. The Norfolk County Asylum at Thorpe is one of the asylums referred to where such arrangements have been made. Here the medical staff will be retained for the treatment and care of the wounded, and this plan is one which seems likely to be generally adopted. The removal of the inmates of the occupied asylums to other institutions might have proved a great hardship to their friends had not care been taken to provide for the issue of free railway passes so that the friends may readily visit the patients without any additional expense. The utilisation of asylum buildings for purposes of military hospitals is a matter of immediate interest, and the questions it raises call for elucidation and discussion.

### TO OUR READERS.

Contributions are specially invited from any of our readers to these columns. They should deal with topical subjects and news. They must be authenticated for the information of the Editor only. The minimum payment if published is 5s. There is no hard-and-fast rule as to space, but notes of about twenty lines in length are preferred.

